# Effects of Hydro‐Ethanolic Extract of *Portulaca oleracea* (Purslane) on Depression, Anxiety, and Stress Symptoms in Patients With Non‐Alcoholic Fatty Liver Disease: A Randomized Double‐Blind Controlled Trial

**DOI:** 10.1002/hsr2.72681

**Published:** 2026-06-19

**Authors:** Narges Milkarizi, Hanieh Barghchi, Mona Nematizade, Saba Belyani, Hossein Bahari, Farnood Rajabzade, Seyede Yegane Ghelichi Kheyrabadi, Maryam Razavidarmian, Mohsen Nematy

**Affiliations:** ^1^ Department of Nutritional Sciences, Faculty of Medicine Mashhad University of Medical Sciences Mashhad Iran; ^2^ Student Research Committee Mashhad University of Medical Sciences Mashhad Iran; ^3^ Department of Nutrition Science Varastegan Institute for Medical Sciences Mashhad Iran; ^4^ Department of Human Sciences, Human Nutrition Program The Ohio State University Columbus Ohio USA; ^5^ Department of Radiology, Mashhad Medical Sciences Branch Islamic Azad University Mashhad Iran; ^6^ Metabolic Syndrome Research Center Mashhad University of Medical Sciences Mashhad Iran

**Keywords:** anxiety, clinical trial, depression, non‐alcoholic fatty liver disease, purslane

## Abstract

**Introduction:**

Non‐alcoholic fatty liver disease (NAFLD) is a prevalent chronic liver condition globally. Managing anxiety, depression, and stress is crucial in NAFLD care. Preliminary hypotheses suggest that *Portulaca oleracea* extract may improve mental health by modulating stress‐related hormones. The high flavonoid content and antioxidant characteristics may diminish inflammation and oxidative stress, which are principal factors contributing to both neuropsychological disorders and NAFLD‐related mental health issues. This study assessed changes in depression, anxiety, and stress levels using the DASS‐21 scale in NAFLD patients after 8 weeks of supplementation with *Portulaca oleracea* extract.

**Methods:**

In a randomized clinical trial, 70 patients were divided into two groups: an intervention group (*n* = 35) and a placebo group (*n* = 35). The intervention group was administered 700 mg of *P. oleracea* extract daily for 8 weeks, whereas the control group received a placebo. Each participant received a calorie‐restricted diet and directives for physical activity. Mental well‐being was assessed with the Depression, Anxiety, and Stress Scale (DASS‐21). Dietary consumption was documented by 3‐day recalls both before and following the intervention.

**Results:**

The average age of the participants was 44.01 ± 8.6 years, with 34 (48.6%) being women. There were significant differences in weight and waist circumference in the intervention group as opposed to the group that received a placebo (*p* < 0.001). Depression and stress scores in the intervention group changed significantly compared to those in the study's placebo group, even after controlling for possible causes (*p* = 0.002, 0.05, respectively). However, before and after controlling for variables including weight and physical activity, there were no appreciable variations in the two groups' anxiety scores (*p* = 0.1 and 0.12, respectively).

**Conclusion:**

Our findings suggest that an 8‐week supplementation with *Portulaca oleracea* can help alleviate depression and stress symptoms in patients with NAFLD.

**Trial Registration:** Clinical trial registration code: IRCT20211116053073N1.

## 1 Introduction

Non‐alcoholic fatty liver disease (NAFLD) has become a significant public health concern worldwide. The prevalence of non‐alcoholic fatty liver disease (NAFLD) is rising along with other non‐communicable diseases such type 2 diabetes, cardiovascular disease, cancer linked to obesity and type 2 diabetes, and severe liver diseases like hepatic cirrhosis and hepatic carcinoma [1].

As a result of rising living standards, dietary and lifestyle modifications, and the increasing incidence of psychological disorders, the prevalence of NAFLD has sharply increased in recent years. The worldwide prevalence of NAFLD was 32% in 2022 [2]. In addition, a previous meta‐analysis reported the prevalence of NAFLD in Iran to be 33 in 2022 [3].

Thus, all patients with NAFLD should undergo lifestyle changes and treatment for the underlying metabolic disorders. Patients with biopsy‐proven NASH and fibrosis should receive specific pharmaceutical treatment [4].

In addition, NAFLD is associated with a higher incidence of psychological disorders, such as anxiety and depression. Due to the lack of specific NAFLD treatments at this time, clarifying these relationships may aid in the management and/or prevention of NAFLD in ordinary clinical practice [5]. Remarkably, NAFLD and these common mental health issues are probably linked in an ongoing cycle that perpetuates itself. Improving knowledge regarding mental health in primary and secondary care pathways related to NAFLD and identifying and interrupting this possible vicious loop in clinical practice may be beneficial for NAFLD therapies [6]. In another study, mitochondrial function or antioxidant defenses were investigated for the treatment of mood disorders, which may also be worth it [7].

According to clinical studies, some dietary components and supplements, along with the quality of food, can help prevent or treat symptoms of stress, anxiety, and depression [8]. Dietary intervention is useful for reducing intrahepatic fat levels and NAFLD side effects [9]. Owing to their ability to improve liver lipotoxicity and inflammation, which are the primary factors in the pathophysiology of the disease, supplements that are effective in reducing oxidative stress have received particular attention [8].

The herbaceous succulent annual plant *Portulaca oleracea* L. is a member of the family Portulacaceae. It is widely distributed in tropical and subtropical regions of the world and is commonly referred to as a purslane [10]. Purslane has been widely used in traditional medicine to treat a variety of conditions, including fever; bladder ulcers; gastrointestinal, spleen, kidney, and skin disorders; hepatoprotective [11], antioxidant [12], and serious inflammation of the liver and other organs [13].

Among green leafy plants, purslane extract is the best source of omega‐3 fatty acids, particularly alpha‐linolenic acid (ALA) [14]. Leucine, isoleucine, lysine, methionine, phenylalanine, cysteine, tyrosine, valine, and threonine are among the amino acids it contains, along with antioxidant vitamins such α‐tocopherol, ascorbic acid, β‐carotene, and glutathione [15]. The symptomatology of mood disorders may be impacted by deficiencies in essential amino acids involved in inflammation, protein synthesis, metabolism, and brain neurotransmission [16].

Several mechanisms have been proposed for the potential mental health benefits of *Portulaca oleracea* L., especially in patients with metabolic disorders such as NAFLD. This plant is good at controlling lipid profiles and has anti‐inflammatory and antioxidant qualities.

These measures could lessen systemic inflammation and oxidative stress, two pathophysiological elements thought to be common to both mood disorders and non‐alcoholic fatty liver disease. Additionally, improvement in mitochondrial function, modulation of inflammatory cytokines, and improvement of antioxidant immune systems may all work together to aid in these individuals' psychological issues [17].

However, despite the promising biological properties of *Portulaca oleracea* L., evidence from clinical studies focusing on its effects on mental health in NAFLD patients remains limited. Most available studies have evaluated its impact on hepatic markers or other metabolic parameters, while the psychological outcomes are less explored. Therefore, further investigation is warranted to clarify the extent of these effects and to determine whether *Portulaca oleracea* L. can be effectively utilized as a complementary treatment for improving mental well‐being in this patient population.

One study showed that patients with NAFLD who paired a weight‐loss diet with 300 mg of *Portulaca oleracea* hydroalcoholic extract every day for a 12‐week period were able to lower inflammation [18]. When physiological pathways are dysregulated, oxidative stress occurs, that leads to neuroinflammation and the eventual emergence of an anxiety disorder, which is also noteworthy [19].

Additionally, research has demonstrated the preventive and therapeutic benefits of purslane polyphenols in neurological conditions [20]. Nevertheless, research on the impact of *Portulaca oleracea* supplementation on NAFLD has improved mental health and quality of life is still lacking. Therefore, this study aimed to determine how the hydroethanolic extract of Portulaca oleracea's aerial parts affected the symptoms of stress, anxiety, and depression among individuals having non‐alcoholic fatty liver.

## 2 Materials and Methods

### 2.1 Study Design

Between March and September of 2022, a parallel‐group clinical trial that was randomized, double‐blind, and placebo‐controlled was carried out. The Mashhad University of Medical Sciences Ethics Committee accepted the study protocol, which complied with the Consolidated Standards of Reporting Trials (CONSORT) (approval code: IR.MUMS.REC.1400.223). The trial protocol was previously published [21] and recorded at the Iranian Clinical Trials Registry (IRCT20211116053073N1).

### 2.2 Participants or Patients' Selection

**Figure 1:**
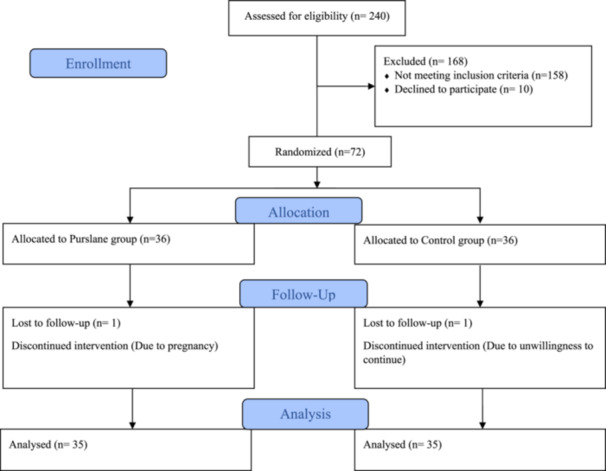
Consort flow diagram.

Seventy NAFLD patients with NAFLD were enrolled in this study (Figure 1). Adults (18–65 years old) with a fatty liver graded F0 or F1 and a verified diagnosis of hepatic steatosis, along with or without fibrosis via sonography, fibroscan, or elastography were necessary to meet the study's inclusion criteria. Pregnancy or nursing, morbid obesity, alcohol consumption greater than 20 g for women and 30 g for men, a history of cancer, liver or renal failure, autoimmune diseases, HIV/AIDS, food allergies to *Portulaca oleracea*, the use of herbal supplements, and the use of hepatotoxic drugs like sodium valproate were the exclusion criteria. Following randomization, patients were excluded if (1) requested by the patient or doctor due to a significant change in the patient's health, (2) exclusion criteria were established, or (3) the patient was sensitive to supplements.

Estimates of sample size were derived from the Soleimani et al. study [22], indicating a mean increase in hepatic steatosis of approximately 65% in the treatment group, whereas the control group's changes were steady or as high as 27%. With a research power of 80%, the sample size was calculated to be 26 individuals per group utilizing the formula for evaluating two amounts of qualitative real estate from two separate statistical societies (*α* = 0.05, *β* = 0.2). To account for potential dropout, a maximum of 15% was considered for each group of 30 patients.

### 2.3 Randomization and Blinding

Using quadruple blocks and sealed envelopes, all participants were categorized into either the placebo or intervention groups based on gender (whether they are male or female) and age (from 18 to 40 and 40 to 65 years old). This helped minimize heterogeneity within the study groups. An independent researcher who was not engaged in the recruitment or intervention processes created the randomization sequence using a computer‐based random number generator. Opaque, sealed, sequentially numbered envelopes were used to guarantee allocation concealment.

### 2.4 Procedures

I. Dose selection

Based on Zheng Guoyin et al.'s investigation, the *P. oleracea* extract dose was calculated employing the following formula: Human equivalent dose (mg/kg) = animal dose (mg/kg) × (Animal Km/Human Km) to convert the effective dose seen in rats to a safe human dose. The resulting 700 mg daily dose (in two capsules) was trialed on five individuals to ensure safety and efficacy. To ensure the safety and tolerability of the selected dose, a short‐term pilot study was conducted on five individuals who received 300 mg of *Portulaca oleracea* extract daily for 1 week. Participants were monitored for any adverse effects, and no significant side effects or intolerance symptoms were reported. This preliminary assessment confirmed that the dose is both safe and well‐tolerated for short‐term use, supporting its application in the main clinical trial.

II. Preparation of capsules

To produce a 70% hydroalcoholic extract, about 350 kg of *P. oleracea* plants from Mashhad were processed. The extract, standardized for phenol content using the Folin–Ciocalteu method and analyzed by LC‐MS/MS, was precisely dosed (350 mg per capsule) before being blended with Avicel, an inert pharmaceutical excipient used to ensure capsule integrity and uniformity. This formulation allowed for accurate dosing while preserving the extract's purity and efficacy. Identical placebo capsules were also made using Avicel and green food coloring to ensure proper blinding. Visual identical placebo capsules were prepared using Avicel and green food coloring. A total of 4200 placebo and 4200 intervention capsules were prepared, coded A and B for blinding, and distributed with approved labels.

III. Interventions

Over the course of 8 weeks, the intervention group was given 700 mg of *P. oleracea* peel daily (1 capsule of 350 mg after breakfast and 1 capsule of 350 mg following dinner). The placebo group received 700 mg of placebo every day for 8 weeks—one 350 mg pill after breakfast and one after dinner. Each participant was given a Mediterranean diet, a hypocaloric diet consisting of 55% carbohydrates, 30% fat, and 15% protein, as well as guidelines for 150 min of physical exercise per week. Every day, follow‐up messages were sent, and every week, phone calls were made.

IV. Follow‐up

The researcher conducted weekly telephone follow‐ups with the participants in this study to assess their condition. Follow‐up appointments with the participants were planned for the final week of the 30‐day term.

### 2.5 Data Collection

Blood samples, serum separation for CBC‐diff observation, anthropometric indicators, dietary records, the IPAQ physical activity assessment, and the completion of relevant questionnaires were all gathered.

### 2.6 Measurements

#### 2.6.1 Anthropometric Measurement

Before, during, and after the trial, a qualified dietitian measured the participants' height and weight using a conventional stadiometer and a clinical scale (SECA), respectively.

## 3 Dietary Intake

During their first visit, participants were asked to complete a 3‐day meal record that included 2 weekdays and 1 weekend. The Nutritionist IV was used to calculate daily calorie, macronutrient, and micronutrient intake. The 3‐day diet record was examined once again on the second appointment and at the end of the trial.

### 3.1 Depression Anxiety Stress Scales (DASS)

To evaluate participants' mood, we utilized the rigorously validated Depression Anxiety Stress Scale (DASS) [23, 24]. Three subscales, each with seven items assessed on a four‐point Likert scale (from 0 to 3), form this 21‐item survey. On the scale, a score of nine or lower indicates that mood‐related symptoms are absent, while a score of nine or above indicates that depression of some severity is present. Similarly, a score of seven or below indicates the absence of mood‐related symptoms, whereas a score of seven or above signifies some degree of anxiety. Furthermore, a score of 14 or below denotes the absence of mood‐related symptoms, whereas a score exceeding 14 indicates the presence of some degree of stress.

### 3.2 Data Collecting

Data were collected for the study at baseline, 30 days after the intervention, and 60 days after the follow‐up visit. In addition to measuring body mass index, height, weight, and gastrointestinal problems, demographic information was gathered via a questionnaire. The participants completed a dietary record and the International Physical Activity Questionnaire. At the beginning and end of the study period, 10 mL of venous blood were extracted in order to measure biochemical variables. ELISA kits were used to quantify and isolate the serum samples.

### 3.3 Data Management

Within 48 h, the research team produced customized forms, scanned, evaluated, and committed to a local site database. The completed forms were stored in a secured cabinet, and data cleansing was a continuous process. Every day, queries that included date, range, and logic checks were generated based on the data stored in a Microsoft Access database.

### 3.4 Statistical Analysis

Statistical analyses were performed using SPSS version 16 (SPSS Inc., Chicago, IL, USA). The Shapiro–Wilk test was used to assess the normality of the variables. Descriptive statistics are presented as mean ± standard deviation (SD) for normally distributed variables and as median (interquartile range, IQR) for skewed distributions. Categorical variables are reported as frequencies and percentages. To compare within‐group changes, paired *t*‐tests were used for normally distributed variables, and the Wilcoxon signed‐rank test was used for non‐normally distributed data. Changes within groups were assessed using paired *t*‐tests. *χ* ^2^ tests were used to compare categorical variables, and independent sample *t*‐tests were used to compare continuous variables between groups (expressed as mean ± SDs). Analysis of covariance (ANCOVA) was conducted to control for covariates, including baseline values, weight, BMI, and physical activity levels, to minimize the influence of potential confounding variables. Partial eta squared (*η* ^2^) was calculated as a measure of effect size for ANCOVA results. Missing data were handled using complete‐case analysis, and no imputation was performed. All tests were two‐tailed, and a *p* value < 0.05 was considered statistically significant. In addition, although ANCOVA was the primary method used to control for confounders, future analyses may benefit from more advanced statistical models such as mixed‐effects models or hierarchical linear modeling to better isolate the effects of individual intervention components, particularly diet and supplementation. All statistical procedures were reviewed by a biostatistician.

### 3.5 Ethical Considerations

The study protocol was reviewed and approved by the Ethics Committee of Mashhad University of Medical Sciences (Approval Code: IR. MUMS. REC.1400.223). All procedures involving human participants were conducted in accordance with the ethical standards of the institutional and national research committee, as well as the 1964 Declaration of Helsinki and its later amendments. Written informed consent was obtained from all participants prior to enrollment in the study.

## 4 Results

### 4.1 Demographic and Clinical Characteristics of the Population

The average age of the 70 participants in this study was 43.1 ± 8.6 years. The purslane and placebo groups had identical age distributions, with mean ages of 43.8 ± 7.6 and 44.2 ± 9.6 years, respectively. No significant age differences were observed (*p* = 0.85). The sample comprised 36 men (51.4%) and 34 women (48.6%). Within the purslane group, 18.5% of the participants had a normal body mass index (BMI), 42.9% were overweight (25 ≤ BMI < 30), and 51.4% were obese, defined as BMI ≥ 30. In contrast, 42.9% of participants in the placebo group were overweight, and 57.1% were obese.

No statistically significant difference was observed between the two groups (*p *= 0.22). The low (less than 600 METs), medium (600 METs–3000 METs), and high (more than 3000 METs) physical activity levels (PAL) of the research participants were 37.1%, 60%, and 2.9%, respectively. Physical activity levels did not differ statistically significantly between the two groups (*p *= 0.22). Furthermore, there were no significant differences between the two groups in terms of cardiovascular disease, diabetes, hypertension, hyperlipidemia, or educational status (*p *> 0.05). The study population's clinical and demographic features are displayed in Table 1.

**Table 1:** Baseline characteristics of participants.

Characteristics	Purslane group	Placebo group	*p* value
Age (years)	43.8 ± 7.6	44.2 ± 9.6	0.85^a^
Sex, *n* (%)			
Female	17 (48.6)	17 (48.6)	1.00
Male	18 (51.4)	18 (51.4)	
PAL, *n* (%)			
Mild	13 (37.1)	16 (45.7)	0.76
Moderate	21 (60)	18 (51.4)	
High	1 (2.9)	1 (2.9)	
BMI (kg/m^2^), *n* (%)			
Normal	2 (5.7)	0 (0)	0.22
Overweight	15 (42.9)	20 (57.1)	
Obese	18 (51.4)	15 (42.9)	
Type 2 diabetes, *n* (%)	2 (6.3)	4 (11.4)	0.56
CVD, *n* (%)	2 (6.1)	3 (8.6)	0.71
Hyperlipidemia, *n* (%)	13 (40.6)	11 (31.4)	0.61
Hypertension, *n* (%)	5 (15.2)	6 (17.1)	0.67
Smoker, *n* (%)	3 (8.6)	7 (20)	0.37

*Note:* Data is presented as mean ± SD or number (percent).

*p* extracted from *χ* ^2^ except (a), which is obtained from 2 independent sample *t‐*test.

*p* < 0.05 is considered statistically significant.

### 4.2 Dietary Intake and Weight Changes Before and After Intervention

The two groups' baseline and post‐intervention dietary macronutrient consumption are shown in Table 2. Before and after the research, both the purslane and placebo groups showed a considerable reduction in daily energy and carbohydrate intake (*p *< 0.01). The daily intake of fat and protein, however, did not vary (*p *> 0.1). Additionally, following the trial, there were no discernible variations in the energy, carbohydrate, protein, or fat intake of the purslane and placebo groups (*p *= 0.55, 0.91, 0.94, and 0.83, respectively). Interestingly, both groups lost weight, but the purslane group outperformed the placebo group in terms of weight reduction by a statistically significant margin (*p *< 0.001) (Table 3).

**Table 2:** Energy, macronutrient, and micronutrient intake at baseline and at the end of Week 8.

Dietary variables	Baseline	After 8 weeks	*p* value^a^	Change	*p* value^b^
Total energy (kcal)					
Placebo	2524.83 ± 318.82	1899.27 ± 301.46	< 0.001	−625.56 ± 131.38	0.55
Purslane	2466.74 ± 294.10	1820.51 ± 264.75	< 0.001	−646.23 ± 133.53	
Protein (g)					
Placebo	87.90 ± 18	82 ± 21.10	0.26	−5.80 ± 26.90	0.94
Purslane	89 ± 24.10	82.60 ± 16.50	0.22	−6.40 ± 26.50	
Carbohydrate (g)					
Placebo	352.20 ± 59.30	317.90 ± 61.40	0.01	−34.30 ± 66.30	0.91
Purslane	374.50 ± 52.60	341.50 ± 61.10	0.001	−32.90 ± 37	
Fat (g)					
Placebo	71.09 ± 25.90	66.45 ± 23.30	0.38	−4.64 ± 27.10	0.83
Purslane	73.80 ± 22	67.30 ± 19.70	0.20	−6.50 ± 22.80	
Fiber (g)					
Placebo	18.70 ± 5.70	16.20 ± 6.30	0.12	−2.50 ± 8.30	0.34^c^
Purslane	18.37 ± 4.10	17.70 ± 4.60	0.54	−0.67 ± 5.90	

*Note:* Data is presented as mean ± SD.

*p* < 0.05 is considered statistically significant.

^a^ *p* is extracted from paired *t‐*test.

^b^ *p* is extracted from 2 independent sample *t‐*test.

^c^ *p* is extracted from Mann–Whitney.

**Table 3:** Anthropometric measurements at baseline and at the end of the intervention.

Variables	Baseline	Week 4	Week 8	Change	*p* value (group)	*p* value (time)	*p* value (group/time)	*p* value^a^
Weight (kg)								
Placebo	88.9 ± 13	87.9 ± 13	87.5 ± 13	−1.4 ± 1.9	0.17	< 0.001	< 0.001	< 0.001
Purslane	85.9 ± 11	84.1 ± 11	82.1 ± 11	−3.8 ± 2.4				
Waist circumference (cm)								
Placebo	108.4 ± 7.9	107.1 ± 8	106.3 ± 8	−2.08 ± 3	0.21	< 0.001	< 0.001	< 0.001
Purslane	107.9 ± 8.9	104.2 ± 9	101.9 ± 9	−5.98 ± 3				
BMI (kg/m^2^)								
Placebo	30.5 ± 4	30.2 ± 4	30 ± 4	−0.52 ± 0.7	0.89	< 0.001	< 0.001	< 0.001
Purslane	30.7 ± 4	30.1 ± 4	29.4 ± 4	−1.37 ± 0.9				

*Note:* Data are presented as mean ± SD.

*p* is extracted from repeated measures.

*p* < 0.05 is considered statistically significant.

^a^ *p* is presented as an adjusted model by weight and physical activity with ANCOVA.

### 4.3 Depression, Anxiety, and Stress Scale Before and After Intervention

In the current study, the mean differences (95% CI) between the purslane and placebo groups post‐intervention were 1.70 (1.30–2.10) for depression, 0.70 (0.16–1.24) for anxiety, and 1.60 (0.75–2.45) for stress, respectively. Depression scores declined in both groups, but only in the Purslane group (*p *= 0.03), and after controlling for potential confounders, the depression score changes were still significant in the purslane group (*p *= 0.003) and more pronounced than in the placebo group (*p *< 0.001). In contrast, anxiety scores decreased in both groups, but these changes were not statistically significant in either the purslane or placebo groups (*p *= 0.07 and 0.08, respectively), and changes in anxiety scores did not differ significantly between the two groups, either before or after controlling for covariates like weight and physical activity (*p *= 0.1 and 0.11, respectively). Interestingly, stress ratings were significantly lower in the purslane group (*p *= 0.02) than in the placebo group (*p *= 0.1). Additionally, both before and after controlling for confounders, including weight and physical activity, the differences in stress scores between the two study groups were determined to be statistically significant (*p *= 0.05). The depression, anxiety, and stress ratings of the two groups prior to and during the intervention are shown in Table 4.

**Table 4:** Depression, anxiety, and stress scale of two groups before and after intervention.

Variable	Purslane		*p* value^a^	Placebo		Mean diff (95% CI)	*p* value^a^	*p* value^b^	*p* value^c^
	Before	After		Before	After				
Depression	10.1 ± 1.2	8.8 ± 0.8	0.03	10.9 ± 1.2	10.5 ± 0.9	1.70 (1.30, 2.10)	0.1	0.003	< 0.001
Anxiety	8.6 ± 0.8	8.0 ± 1.3	0.07	8.9 ± 1.2	8.7 ± 0.9	0.70 (0.16, 1.24)	0.08	0.1	0.11
Stress	11.02 ± 0.5	10.1 ± 1.2	0.02	12.1 ± 1.1	11.7 ± 2.1	1.60 (0.75, 2.45)	0.1	0.05	0.05

*Note:* Data is presented as Mean ± SD. Mean difference (95% CI) represents the unadjusted difference between the two groups at the post‐intervention stage.

*p* < 0.05 is considered statistically significant.

^a^ *p* is extracted from paired *t*‐test.

^b^ *p* is extracted from 2 independent sample *t*‐test.

^c^ *p* is presented adjusted model by weight and physical activity, with ANCOVA.

## 5 Discussion

Our study's main goal was to determine how 60 days of *Portulaca oleracea* supplementation affected the psychological health of NAFLD patients. Our findings showed that during the trial, there were no appreciable differences in the *Portulaca oleracea* and placebo groups' calorie, carbohydrate, protein, or fat intake. Additionally, our assessment using the Depression, Anxiety, and Stress Scale (DASS) revealed a significant reduction in depression and stress symptoms in the *Portulaca oleracea* group following the intervention, although the reduction in anxiety scores did not reach significance when comparing the two groups. These findings suggest that purslane supplementation may offer a safe, non‐pharmacological approach to reducing stress and depression symptoms in NAFLD patients, which could be valuable in clinical settings where psychological support is limited.

Mood problems are more common in those with non‐alcoholic fatty liver disease [25, 26]. Although the precise explanation of this linkage is yet unknown, it could be related to the high correlation between NAFLD and diabetes mellitus and insulin resistance, all of which have been connected to feelings of anxiety and depression [27, 28]. It is crucial to remember that variables like being overweight or not following diabetic treatment regimens are not the only causes of the association between diabetes mellitus, insulin resistance, and mood problems [27, 28, 29]. The fact that psychological disorder treatments, such as psychotherapy and antidepressants, have been shown to improve insulin resistance and glycemic control without regard to weight loss or adherence to diabetes therapy lends credence to this [30, 31, 32]. In contrast, elevated levels of cortisol, epinephrine [33], and pro‐inflammatory cytokines in individuals with mood disorders [34, 35, 36] may present another plausible explanation for the association between these mood disorders and NAFLD.

NAFLD patients with or without serious mental disorders had inadequate medical responses, according to 48‐week lifestyle intervention research [37]. Patients with depressed NAFLD have been shown to have poor responses to therapy due in part to memory and self‐efficacy problems. Consequently, a better prognosis may result from treating depression simultaneously in NAFLD patients who also have depressed symptoms.

Abdominal obesity, diabetes, and chronic systemic inflammation are among the risk factors that are prevalent to depression and non‐alcoholic fatty liver disease (NAFLD) [38]. The degree of depression in NAFLD patients has also been linked to lifestyle variables, such as body mass index and hypertension [39]. Consequently, treatment of NAFLD and weight loss may have a beneficial impact on the management of NAFLD‐related depression. Our study shows that weight reduction in NAFLD patients with NAFLD was accompanied by an improved psychological state, as indicated by the better BMI and waist circumference observed in our results following the intervention.

The significance of stress management in the prevention and treatment of NAFLD was highlighted by a 2020 study that found a 1.3‐fold greater risk of NAFLD in people with higher stress symptoms [40]. Furthermore, the importance of stress management in the treatment of nonalcoholic fatty liver disease is further highlighted by the higher hepatic disease mortality in those who experience emotional difficulties [41]. The *Portulaca oleracea* group in our 8‐week supplementation trial had a lower stress level, which improved their responsiveness to the medication.

The medicinal properties of purslane and its primary constituents are numerous and include anti‐inflammatory [42], antioxidant [43, 44], immunomodulatory [45], antidiabetic [46], antimicrobial [47], anticancer [48], neuroprotective [49, 50], antidepressant [51], and anxiolytic qualities [52]. Its possible antidepressant qualities are supported by its rich mineral content, which includes lithium, folate, calcium, potassium, and magnesium [53]. Up to 16% of the plant's dry weight is made up of antidepressant compounds [54].

Purslane has been found to reduce immobility time [51] and adrenocorticotropic hormone (ACTH) levels, which are associated with the induction of postpartum depression, comparable to diazepam. This suggests that purslane exerts its antidepressant effect by reducing ACTH levels [55, 56].

In vivo studies in mice have shown that purslane has anxiolytic effects, as indicated by increased time spent in the open arms and decreased anxiety reactions. Purslane could exhibit anxiolytic effects via influencing the GABAergic system [52, 57].

Moreover, oxidative stress and inflammation are significant underlying causes of these individuals' mental illnesses as well as NAFLD. *Portulaca oleracea's* anti‐inflammatory effects of *P. oleracea* may be attributed to its flavonoid‐rich extracts, which inhibit the expression of iNOS and COX‐2 and partially suppress NF‐κB and MAPK activation in LPS‐stimulated cells [58]. This is consistent with data showing that dietary flavonoids have anti‐inflammatory properties in both in vitro and in vivo settings [59, 60]. Furthermore, steatosis, liver fibrosis, and cognitive problems are all significantly influenced by oxidative stress, which is caused by an imbalance between free radicals and the body's antioxidant system [61, 62]. Free radicals can interfere with insulin signaling pathways, damage mitochondrial structure, decrease hepatocyte beta‐oxidation of free fatty acids, trigger inflammatory cascades, and eventually increase the distribution of free fatty acids to the liver. Supplementing with *Portulaca oleracea* may therefore lessen oxidative stress, suppress the pro‐inflammatory process, and delay the onset of stress and mental illnesses [63, 64].

Previous research found that *P. oleracea* might prevent the oxidative stress response by lowering malondialdehyde (MDA) and nitric oxide (NO) levels in mice colitis caused by dextran sulfate sodium [65]. NO and MDA levels in the testis, kidney, and liver of rats treated with the *P. oleracea* aqueous extract for 12 days were significantly reduced [66].

*P. oleracea* might reduce the base asset processes of sadness, anxiety, and stress associated with non‐alcoholic fatty liver disease (NAFLD) by decreasing oxidative stress, gut microbiota dysbiosis, and insulin resistance associated with obesity. To ascertain the exact mechanisms behind the management of mental diseases in NAFLD, more investigation is necessary.

## 6 Limitations

There are a number of limitations to this study that should be noted. The results' statistical power and applicability to the larger NAFLD population may be impacted by the small sample size. The intervention period may have been insufficient to observe long‐term effects of *Portulaca oleracea* supplementation. Additionally, the reliance on self‐reported measures for psychological outcomes such as depression and stress could introduce response bias. The results might have been affected by potential confounding variables that were not completely controlled, such as nutritional consumption, levels of physical activity, and other lifestyle characteristics. Future studies with larger, more diverse populations, longer follow‐up periods, and controlled lifestyle variables are recommended to validate and expand upon these findings.

## 7 Conclusion

Our research concludes that people with non‐alcoholic fatty liver disease could benefit from using *Portulaca oleracea* supplements. It is linked to weight loss in addition to a decrease in feelings of stress and despair. Addressing psychological well‐being and stress management may be crucial for NAFLD treatment. Although *Portulaca oleracea* shows promise, further research is needed to understand its precise mechanisms and role in managing mental health in NAFLD. Future studies should also investigate different dosages and durations of supplementation, as well as their potential effects on other aspects of mental and physical health.

## Author Contributions

**Narges Milkarizi:** conceptualization, investigation, data curation, and formal analysis. **Hanieh Barghchi:** investigation and data curation. **Mona Nematizade:** writing – original draft. **Saba Belyani:** writing – original draft. **Hossein Bahari:** writing – review and editing. **Farnood Rajabzade:** resources and supervision. **Seyede Yegane Ghelichi Kheyrabadi:** writing – original draft. **Maryam Razavidarmian:** software. **Mohsen Nematy:** conceptualization, funding acquisition, validation, project administration, and methodology.

## Ethics Statement

Every experiment was carried out in compliance with Mashhad University of Medical Sciences' and the Declaration of Helsinki's ethical standards. The Mashhad University of Medical Sciences School of Medicine Biomedical Research Ethics Committee gave its approval to the study protocol (IR.MUMS.REC.1400.223). Prior to taking part in the trial, each subject gave written informed consent.

## Conflicts of Interest

The authors declare no conflicts of interest.

## Data Availability

The data that support the findings of this study are available on request from the corresponding author. The data are not publicly available due to privacy or ethical restrictions.
